# Effects of low dose silver nanoparticle treatment on the structure and community composition of bacterial freshwater biofilms

**DOI:** 10.1371/journal.pone.0199132

**Published:** 2018-06-14

**Authors:** Alexandra Y. Grün, Constantin B. App, Andreas Breidenbach, Jutta Meier, George Metreveli, Gabriele E. Schaumann, Werner Manz

**Affiliations:** 1 Institute for Integrated Natural Sciences, University of Koblenz-Landau, Koblenz, Germany; 2 Institute for Environmental Sciences, Group of Environmental and Soil Chemistry, University of Koblenz-Landau, Landau, Germany; VIT University, INDIA

## Abstract

The application of engineered silver nanoparticles (AgNPs) in a considerable amount of registered commercial products inevitably will result in the continuous release of AgNPs into the natural aquatic environment. Therefore, native biofilms, as the prominent life form of microorganisms in almost all known ecosystems, will be subjected to AgNP exposure. Despite the exponentially growing research activities worldwide, it is still difficult to assess nanoparticle-mediated toxicity in natural environments. In order to obtain an ecotoxicologically relevant exposure scenario, we performed experiments with artificial stream mesocosm systems approaching low dose AgNP concentrations close to predicted environmental concentrations. Pregrown freshwater biofilms were exposed for 14 days to citrate-stabilized AgNPs at a concentration of 600 μg l^-1^ in two commonly used sizes (30 and 70 nm). Sublethal effects of AgNP treatment were assessed with regard to biofilm structure by gravimetric measurements (biofilm thickness and density) and by two biomass parameters, chlorophyll *a* and protein content. The composition of bacterial biofilm communities was characterized by t-RFLP fingerprinting combined with phylogenetic studies based on the 16S gene. After 14 days of treatment, the structural parameters of the biofilm such as thickness, density, and chlorophyll *a* and protein content were not statistically significantly changed by AgNP exposure. Furthermore, t-RFLP fingerprint analysis showed that the bacterial diversity was not diminished by AgNPs, as calculated by Shannon Wiener and evenness indices. Nevertheless, t-RFLP analysis also indicated that AgNPs led to an altered biofilm community composition as was shown by cluster analysis and multidimensional scaling (MDS) based on the Bray Curtis index. Sequence analysis of cloned 16S rRNA genes further revealed that changes in community composition were related with the displacement of putatively AgNP-sensitive bacterial taxa Actinobacteria, Chloroflexi, and Cyanobacteria by taxa known for their enhanced adaptability towards metal stress, such as Acidobacteria, Sphingomonadales, and Comamonadaceae. This measurable community shift, even after low dose AgNP treatment, causes serious concerns with respect to the broad application of AgNPs and their potentially adverse impact on the ecological function of lotic biofilms, such as biodegradation or biostabilization.

## 1 Introduction

During the last decade, engineered silver nanoparticles (AgNPs) have become a substantial part of modern commodities, with applications in textiles, personal care products and pharmaceuticals. After use, AgNPs will ultimately reach the aquatic environment and expected elevated levels of AgNPs [1–3] suggest that AgNPs will act as a metal pollutant stress and increasingly affect bacterial communities within these ecosystems. Most of the AgNPs are expected to be sulfidized and accumulated in the sludge of the waste water treatment plants (WWTP), and most probably, only small part can reach the surface waters via direct discharge of WWTP efluents [4–5]. Once released into the river water, AgNPs will undergo rapid aggregation mainly due to the presence of divalent cations [6–7]. Although natural organic matter (NOM) leads to colloidal stabilization of AgNPs at low cation concentrations, NOM can induce the formation of bridging determined aggregates at high cation concentrations [7]. Consequently, the aggregation of AgNPs can finally result in their sedimentation accompanied by accumulation in microbial biofilms [8], which serve as a sink for organic and inorganic pollutants. Due to their metabolic and genetic diversity, bacterial biofilm communities play a key role in the production and degradation of NOM; cycling of nitrogen, phosphorous, sulfur and metals [9]; and transformation and/or degradation of pollutants. Metals and metalloids have significant effects on microorganisms and impair their ecological function [10]. Consequently, an enrichment of AgNPs or their transformation products in freshwater biofilms by contamination or redistribution may lead to comparable toxic effects. Because bactericidal properties of AgNPs are attributed to the release of Ag^+^-ions by oxidative dissolution [11–13], AgNPs might serve as a continuous source for Ag^+^-ions and thus have a long-term impact on biofilms.

Adverse effects of AgNPs on bacteria are generally claimed to be associated with the inhibition of enzyme activities, DNA transcription, and suppression of bacterial respiration [1]. Pertaining to the effects of AgNPs on bacteria in aquatic environments, a range of habitats, such as marine biofilms [14–15], activated sludge [16–17], surface stream water [18], and freshwater biofilms [8, 19], have been investigated. A reduction in biomass [8, 14], alterations in bacterial community composition [14, 17–18] and reduced metabolic activities [18–19] as a consequence of AgNP exposure have been reported. Contradictory effects have been shown for wastewater biofilms which were highly tolerant to AgNP treatment [16] and for marine biofilms which were only negligibly affected [15]. Overall, only a few studies have addressed natural habitats. Freshwater biofilms of streams and rivers are of high interest, as these habitats receive waste effluents of water treatment plants and are most likely to be confronted with AgNPs [8, 19]. In this area there is a critical knowledge gap, which needs to be filled up because of the relevance of freshwater biofilms in important ecosystem functions. The composition of the bacterial community in these freshwater biofilms as well as the structure of the biofilms substantially contribute to the ecosystem functions such as biodegradation with respect to nutrient loads, transformation and/or degradation of pollutants, and biostabilization. Hence, this study aims to broaden our knowledge of the impact of AgNPs on freshwater biofilms of rivers with respect to biofilm structure and bacterial community composition.

As it is still challenging to monitor the ecotoxicity of AgNPs in natural freshwater environments, there is a need for suitable experiments to facilitate this task. Mesocoms studies provide the possibility for the detailed investigation of effects of AgNPs on freshwater biofilms under realistic environmental conditions. In contrast to the studies of Kroll et al. [8] and Gil-Allué et al. [19], in which biofilms from streams were investigated, our mesocosm study addresses artificially lotic biofilms grown and incubated in mesocosms filled with water and sediment from a river. In the present study, lotic biofilms pre-grown in a 500-l mesocosm were translocated in smaller 60-l mesocosms and treated with AgNPs. To approximate environmentally relevant concentrations of Ag NPs, a concentration of 600 μg l^-1^ AgNPs was chosen for use in all experiments. This would be still at the higher end of the concentration range estimated for natural environments, which ranges from 0.1 pg l^-1^ to a few ng l^-1^ in the surfaces of fresh water and up to ~750 μg kg^-1^ in the sediments of fresh water [3]. Thus, 600 μg l^-1^ AgNPs would mimic a worst case scenario, such as the resuspension of sediments by flooding events or production plant outfalls [3]. Because the toxicity of AgNPs is often claimed to be dependent on their size [20–21], we applied citrate-stabilized AgNPs with two different sizes (30 nm and 70 nm). After an exposure time of 14 days, we tested the hypothesis that AgNPs in a low dose concentration affect the biofilm structure in terms of architectural and biomass parameters. Furthermore, an expected alteration of the bacterial community composition induced by AgNPs was characterized by a combination of t-RFLP fingerprinting and sequence analysis of cloned 16S rRNA genes.

## 2 Materials and methods

### 2.1 General experimental setup

**Fig 1:**
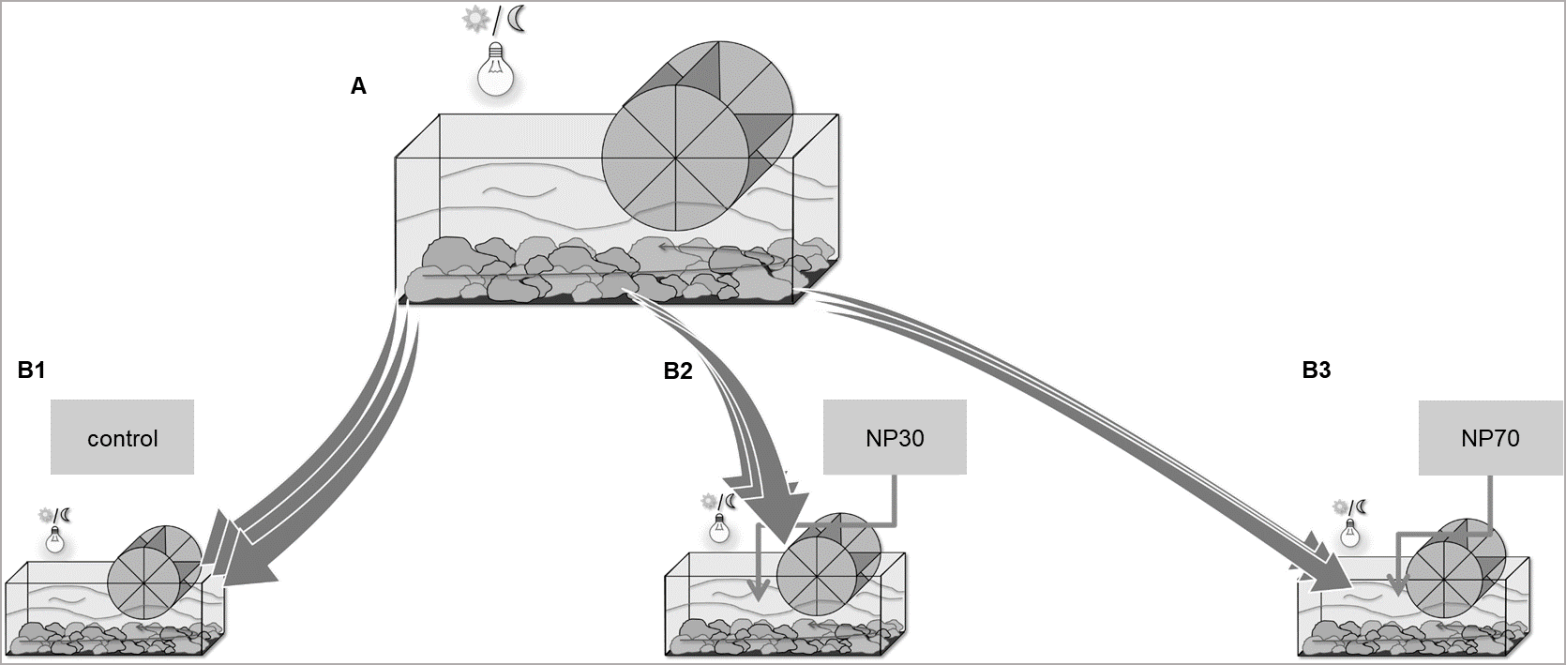
Experimental setup. Lotic biofilms were grown in an indoor stream mesocosm (A) and translocated for exposure treatments into three individual mesocosms (B1, B2, and B3). B1: Control assay without AgNPs; B2: AgNPs 30 nm; B3: AgNPs 70 nm. All experiments were performed in triplicate. The incubation periods of pre-grown biofilms (14 days) (A) as well as the incubation periods for exposure treatments (14 days) (B1-B3) were performed in three consecutive runs without time interruption within 12 weeks.

Bacterial lotic freshwater biofilms were grown for 14 days in an indoor stream mesocosm (Fig 1). The mesocosm (0.5 m high, 0.6 m wide, and 2.2 m long) was initially filled with 500 l of natural river water and 100 kg of sediment (pebble stones) obtained from the river Rhine (stream km 596, Niederwerth, Germany). The stream flow velocity was adjusted to 0.3 m s^-1^ and maintained by an electrically driven paddlewheel. The flow velocity and the physico-chemical parameters, such as temperature, pH-value, conductivity and oxygen concentration, were continuously monitored (Multi 3430 Set F, WTW, Weilheim, Germany). The nutrient concentrations in the stream water (total phosphor, nitrate, nitrite, ammonium) were measured photometrically (Photolab 6600 UV-VIS, WTW, Germany; Spectroquant® Merck, Darmstadt, Germany). Biofilms were grown on sterile microscopic glass slides arranged and locked in a customized slide trail placed on top of the mesocosm sediment. Artificial day light (Osram L 18W/965 Biolux G13, München, Germany) simulated a 12-h/12-h light-dark-cycle in the mesocosm. After biofilms were established, the slide trails were transferred into three independent smaller mesocosms (0.3 m high, 0.3 wide, and 0.6 m long). For treatment with AgNPs, the mesocosms were filled with the identical water (10 L) to sediment (2 kg) ratio obtained from the river Rhine and operated under the same water flow adjusted by centrifugal pumps (Tetra WP 300, Tetra, Melle, Germany). Lotic biofilms were exposed to a single pulse of AgNPs that were 30 nm or 70 nm in size (designated as NP30 and NP70 respectively) at a concentration of 600 μg l^-1^ AgNP, followed by an incubation period of 14 days. All experiments were performed in triplicate.

### 2.2 Synthesis and characterization of AgNPs

A 30 nm sized AgNP dispersion (NP30) was synthesized by citrate reduction according to Metreveli et al. (2015) with a concentration of 100 mg l^-1^. The AgNP dispersion with the particle size of 70 nm (NP70) which was also citrate stabilized, was purchased from Particular (Hannover, Germany) at a concentration of 107 mg l^-1^. Both nanoparticle stock dispersions were kept at +4°C in the dark. The mean particle diameter and particle size distribution of the AgNPs were determined using dynamic light scattering (DLS). All of the details of the AgNP characterization and aggregation behavior have been described previously [22]. AgNP dispersions used in this study showed clearly distinct particle size fractions. The particle size in the NP30 dispersion was 32 ± 3 nm (mean ± standard deviation) (DLS), with a polydispersity index of 0.40 ± 0.02 (mean ± standard deviation), and the particle size in the NP70 dispersion was 97 ± 3 nm (mean ± standard deviation) (DLS), with a polydispersity index of 0.20 ± 0.01 (mean ± standard deviation). Irrespective of the differences between the nominal and measured particle sizes for NP70 we will call this dispersion as AgNPs with the particle size of 70 nm as declared by manufacturer (more information see Grün et al.[22]).

### 2.3 Analyses of biofilms

Quantitative biofilm parameters were determined by gravimetric measurements following the procedure of Staudt et al. [23]. Glass slides with lotic biofilms for each experiment were drained in a vertical position for 5 min, and subsequently, the wet weight was determined. The dry weight of the biomass was determined after heat treatment at 65°C for 24 h. Dried biomass was also used for subsequent protein measurements. To calculate the wet (*m_WF_*) and dry masses (*m_DF_*), the tare weight of the slides was determined separately. Biofilm thickness (*L_F_*) was calculated from the wet mass (*m_WF_*) and the surface area of the slide (*A_F_* = 19.76 cm^2^) assuming a density of wet biofilm (ρ_*WF*_) of 1 g cm^-3^ using the following equation

$$L_{F}=\frac{m_{WF}}{\rho_{WF}∙A_{F}}$$ (1)

The mean biofilm density (ρ_*F*_) was calculated from:

$$\rho_{F}=\frac{m_{DF}}{\frac{m_{WF}}{\rho_{WF}}}$$ (2)

For weighing, a balance with 5 digit accuracy was used. Each measurement was performed with four replicates.

The protein content of the biofilms was analyzed using dried biofilm material. Material from 14-day-old pre-grown biofilms was scraped off from four glass slides with a total area of 79.04 cm^2^, and material from 28-day-old control and AgNP-exposed biofilms was each scraped off from two glass slides with a total area of 39.52 cm^2^. Biofilm material from 14-day-old pre-grown biofilms was taken from a higher surface area to provide an adequate amount of biofilm material for protein measurements and determination of chlorophyll *a*. The resulting biofilm material was disrupted following modifications of the procedures given by Chisti [24] and Cheng [25]. Biofilm material was subsequently suspended in 1 ml of sterile Milli-Q water. After centrifugation for 2 min at 16,600 × *g*, the biomass was treated with 1 ml of 0.5 M NaOH, followed by a further incubation step for 1 h at 95°C, and sonicated for 45 min. The supernatant containing the soluble protein fraction was obtained after a final centrifugation for 30 min at 18,300 × *g* at 4°C. One milliliter of the BC-Assay Protein Quantification Kit reagent (Uptima, Montluçon, France) was added to 50 μl of the supernatant, and the total protein content of the biofilms was measured photometrically following the manufacturer’s instructions with bovine serum albumin as a protein concentration standard. The measurements for each experiment were performed in duplicate, and the experiment was performed in triplicate. Data were normalized to μg per cm^2^ biofilm surface.

The determination of chlorophyll *a* was performed following a modification of the procedure given by Mewes et al [26]. Wet biofilm material taken from either a 79.04 cm^2^ glass slide surface area of 14-day-old pre-grown mesocosm biofilms or taken from a 39.52 cm^2^ glass slide surface area of 28-day-old control and AgNP-exposed biofilms was pooled. Material was transferred into 1 ml of sterile Milli-Q water followed by centrifugation for 2 min at 16,600 × *g*. The resulting biofilm pellet was resuspended in 1 ml of 96% ethanol buffered with 1 g l^-1^ MgCO_3_, followed by sonication for 15 min. After incubation for 3 h in the dark, the biofilm material was centrifuged for 2 min at 16,600 × *g*. Absorbance of the supernatant was measured at wavelengths of 665 nm and 750 nm using a spectrophotometer (SPECORD 200 Plus Edition 2010, Analytik Jena, Jena, Germany). The chlorophyll *a* concentration (μg cm^-2^) was calculated after turbidity correction at 750 nm using the following equation

$${Chl}_{a}(\mu g\;{cm}^{-2})=\frac{\varepsilon *(E665-E750)*Ve}{\left(Vf*d)*(\frac{Vg}{A}\right)}$$ (3)

where ε = 8.5213 (*Chl*_*a*_ from spinach, R^2^ = 0.9994); E = absorption at the indicated wavelength (nm); *Vf* = dilution factor, (ml); *Ve* = volume of extraction (ml); *d* = path length of cuvette, 1 cm; *Vg* = total volume of the sample (ml); and *A* = area (cm^2^).

All measurements were performed in duplicate.

Genomic DNA extraction was modified based on the method described by Morán et al. [27]. The biofilm biomass taken from a 98.8 cm^2^ glass slide surface area was pooled and washed in TE buffer (10 mM Tris-HCl, pH 7.4; 1 mM EDTA, pH 8), and the pellet was stored at -20°C until further use. For cell lysis, 400 μl of a 2% cetyltrimethylammonium bromide (CTAB) solution and 3 μl of a proteinase K solution (250 mg ml^-1^) were added. After 1 h of incubation at 60°C, 134 μl of a 10% sodium dodecyl sulfate (SDS) solution was added, followed by a second incubation step at 60°C for 1 h. DNA purification was achieved by phenol-chloroform-isoamylalcohol extraction followed by precipitation in ethanol. Bacterial 16S rRNA genes were amplified from DNA extracts using the universal bacterial primers 27f (5’ AGA GTT TGA TCM TGG CTC AG 3’) and 1492r (5’ TAC GGY TAC CTT GTT ACG ACT T 3’) [28]. For T-RFLP analysis, 27f primers were 5’-end labeled with 6-carboxyfluorescein (Biomers, Ulm, Germany). Amplification was performed by an initial denaturation step at 94°C for 4 min, 30 cycles of 45 s at 94°C, 1 min at 58°C, and 2 min at 72°C, followed by a final extension step at 72°C for 10 min.

For t-RFLP analysis, PCR products obtained in triplicate were pooled and purified (SureClean, Bioline, Luckenwalde, Germany) and subsequently quantified by agarose gel electrophoresis. Approximately 10 ng of DNA was digested using the restriction enzymes *Msp*I and *BstU*I (New England Biolabs, Frankfurt, Germany). DNA fragments were purified by ethanol precipitation and loaded on a capillary sequencer (ABI PRISM® 310 Genetic Analyzer, Applied Biosystems, Weiterstadt, Germany). Map-Marker®1000 (Eurogentec, Seraing, Belgium) was used as an internal standard. Fragment length assignment was carried out with GeneMapper® Software (Applied Biosystems, Weiterstadt, Germany). According to an algorithm developed by Abdo et al. [29], data obtained from t-RFLP fingerprints were trimmed to a min/max fragment length of 50 and 800 bp and the background noise was removed. This was implemented using an R‐script (written by Ingo Fetzer) in R statistical software [30].

Sequence analysis of 16S rRNA genes. PCR products generated in triplicate for each replicate and condition (control, NP30, NP70) were purified using the MinElute PCR Purification Kit, (Qiagen, Hilden, Germany) following the manufacturer’s protocol. Fresh PCR products were ligated into the pDrive Cloning Vector (Qiagen, Hilden, Germany) according to the manufacturer’s protocol and transferred into competent cells of *Escherichia coli* DH5α. Positive clones were checked for the appropriate insert size by M13-PCR and were further screened by amplified ribosomal DNA restriction analysis (ARDRA) using *Msp*I. In total, 275 clones were analyzed. Similar clones were identified by software-assisted band pattern comparison (Phoretix 1D Software, Nonlinear Dynamics, Newcastle upon Tyne, UK). Representative clones of each unique ARDRA pattern were partially sequenced by LGC Genomics (Berlin, Germany) using the 27f primer. Sequences were checked manually for ambiguous codes and primer regions and terminal ambiguous sequences were trimmed off. Chimeric sequences were identified using the DECIPHER software package [31]. The BLASTN program [32–33] was used to check for similar sequences in the GenBank nucleotide sequence database. All cloned 16S rRNA gene sequences were clustered into operational taxonomic units (OTUs) based on i) the ARDRA pattern alone or ii) in combination with sequence analysis. Sequence analysis of representative clones was classified at a 97% identity threshold using the Ribosomal Database Project-II software package release 11.3 [34]. Organelle sequences were excluded from the phylogenetic bacterial community composition analysis by filtering out all of the sequences whose taxonomy was assigned to chloroplasts. All of the determined 16S rRNA gene sequences were deposited in the GenBank nucleotide sequence database under the accession numbers KX977121—KX977301.

### 2.4 Data analysis

Data from gravimetric measurements, the protein and chlorophyll *a* contents, as well as diversity indices (see below), were checked for normality by the Shapiro-Wilk test [35] using the software package R [30]. Differences between treated and untreated biofilms were calculated using the vegan package [36] for R using one-way ANOVA, followed by a Tukey’s posthoc test [35].

The diversity indices, Shannon Wiener index, species evenness and Bray Curtis index were calculated based on the t-RFLP data using the PAST software package [37]. Cluster analysis and MDS plotting were performed based on the Bray Curtis index. Based on OTUs, the Shannon, Alpha and Simpson diversities were determined and a rarefaction curve was calculated using the software biodiversity pro [38].

## 3 Results

### 3.1 Biofilm structure in response to AgNP exposure

The physical and chemical water parameters remained constant with only slight variation between the treatments and their replicates (S1 Table). The nutrient concentrations in water were constant over the whole experimental period with phosphate in the range below 0.05 mg l^-1^, nitrate below 0.5 mg l^-1^, nitrite below 3 mg l^-1^, and ammonium below 4 mg l^-1^. Hence, stable water parameters allowed for interpretation of the data obtained from the analysis of the structure and bacterial community composition of the lotic biofilms as a consequence of applied AgNPs.

**Fig 2:**
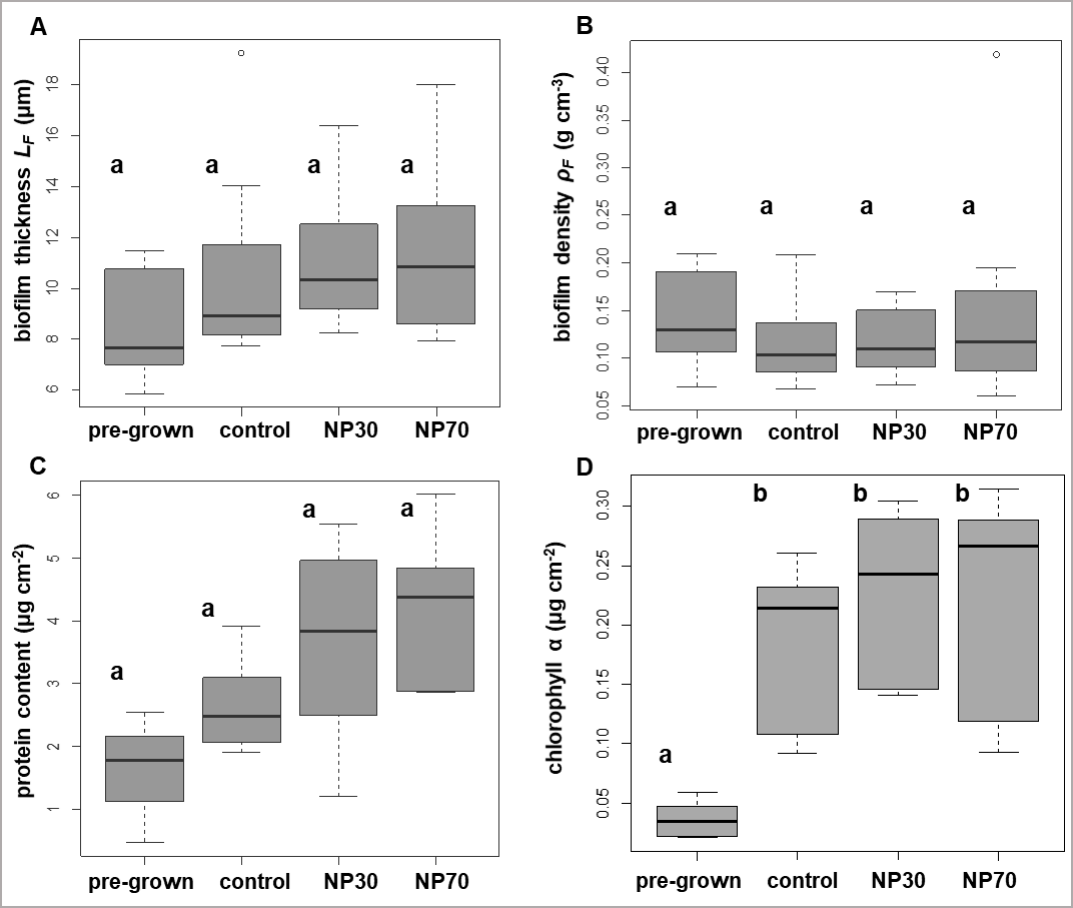
Architectural and biomass parameters of biofilms. Biofilm thickness *L_F_* (μm), n = 12 (A); biofilm density ρ_*F*_ (g cm^-3^), n = 12 (B); protein content (μg cm^-2^), n = 6 (C); chlorophyll *a* content (μg cm^-2^), n = 6 (D). Stripes show the medians, boxes the inter quartiles, the dots the outliers and the whiskers extend to the extremes. Different letters denote statistically significant differences. The values with the same letters are not statistically different. (p < 0.05; one-way ANOVA, Tukey’s posthoc test).

The biofilm thickness (*L*_*F*_) increased from 8.5 ± 2.1 μm (mean ± standard deviation) in pre-grown biofilms (14 d) to 10.5 ± 3.4 μm, 11 ± 2.4 μm, and 11 ± 3.1 μm in control, NP30-, or NP70-treated biofilms, respectively, (28 d), but the increase was not statistically significant (p > 0.05) (Fig 2A). Treatments with NP30 or NP70 did not show significant effects compared to the controls.

The biofilm density (*ρ*_*F*_) was similar for all analyzed biofilms. The density of pre-grown biofilms was 0.14 ± 0.05 g cm^-3^, and control, NP30-, and NP70-treated biofilms had densities of 0.11 ± 0.04 g cm^-3^, 0.12 ± 0.03 g cm^-3^, and 0.15 ± 0.1 g cm^-3^, respectively. No statistically significant differences occurred among the different treatments (p > 0.05; control, NP30, NP70) (Fig 2B).

The protein content increased from 1.60 ± 1.04 μg cm^-2^ in pre-grown biofilms to 2.65 ± 0.58 μg cm^-2^, 3.64 ± 1.71 μg cm^-2^, and 4.22 ± 1.29 μg cm^-2^ in control, NP30-, and NP70-treated biofilms, respectively (Fig 2C). This increase was not significantly different compared to pre-grown biofilms (p > 0.05). Treatments with NP30 or NP70 did not evoke significantly different values for protein contents compared to the control (p > 0.05) (Fig 2C).

The chlorophyll *a* contents of control, NP30-, and NP70-treated biofilms ranged between 0.19 ± 0.01 μg cm^-2^, 0.23 ± 0.07 μg cm^-2^, and 0.22 ± 0.09 μg cm^-2^, respectively, and were significantly elevated compared to the chlorophyll *a* content of pre-grown biofilms, with a content of 0.04 ± 0.01 μg cm^-2^ (p < 0.05) (Fig 2D). Within 28 d biofilms, exposure to NP30 or NP70 did not significantly alter the chlorophyll *a* contents compared to untreated control biofilms (p > 0.05) (Fig 2D).

In summary, the architectural and biomass parameters were not statistically significantly affected by AgNP exposure.

### 3.2 Biofilm community composition and diversity

t-RFLP analysis revealed that AgNPs of both sizes had a distinct influence clearly distinguishable from untreated biofilms on the composition of bacterial communities of observed biofilms, whereas the bacterial diversity was not negatively impacted by AgNPs.

The number of obtained terminal restriction fragments (t-RFs) was relatively similar among the treatments and replicates, resulting in 29–36 t-RFs after digestion with *Msp*I or *BstU*I (Table 1). A lower number of t-RFs was consistently found in control biofilms, with 10 (*Msp*I) or 8 (*BstU*I) specific fragments. 42 (*Msp*I) or 24 (*BstU*I) distinct t-RFs were present only in the NP30 and NP70 treatments.

**Table 1:** Number of t-RFs.

		control				NP30				NP70			
replicate		1	2	3	Ø	1	2	3	Ø	1	2	3	Ø
**fragments**	*Msp*I	20	28	39	**29**	27	32	45	**35**	34	28	39	**34**
	*BstU*I	28	33	39	**33**	36	34	33	**34**	37	29	43	**36**
**Shannon Wiener**	*Msp*I	2.17	2.89	3.15	**2.74**	2.78	3.07	3.42	**3.09**	3.07	2.98	3.43	**3.16**
	*BstU*I	2.37	2.88	2.98	**2.74**	3.03	2.9	2.91	**2.95**	3.0	2.91	3.42	**3.11**
**evenness**	*Msp*I	0.44	0.64	0.60	**0.56**	0.60	0.67	0.68	**0.65**	0.63	0.70	0.80	**0.71**
	*BstUI*	0.38	0.54	0.50	**0.47**	0.58	0.53	0.56	**0.56**	0.54	0.63	0.71	**0.63**

Number of t-RFs after digestion with *Msp*I or *BstU*I and the corresponding values for the Shannon Wiener index and evenness.

Ø, mean; Calculated Shannon Wiener indices and evenness indices obtained from digestion with *BstU*I compared well with those for *Msp*I.

Two general responses were observed by AgNP exposure after calculating the Shannon Wiener indices and evenness indices. Biofilm communities treated with AgNPs indicated a higher bacterial phylogenetic diversity compared to control biofilms. The Shannon Wiener indices for NP30- or NP70-treated biofilms were 3.09 or 3.16, respectively, and were increased compared to those of the control biofilms, which had an index of 2.74. The evenness indices revealed a more equally distributed species abundance in the biofilm communities treated with NP30 or NP70. The evenness indices increased from the control (0.56) to treatments with NP30 (0.65) and NP70 (0.71). However, based on these data, the detectable differences between the control and treatments were not significant. Likewise, no significant differences occurred between the NP30 and NP70 treatments (p > 0.05; one-way ANOVA, Tukey’s posthoc test).

**Fig 3:**
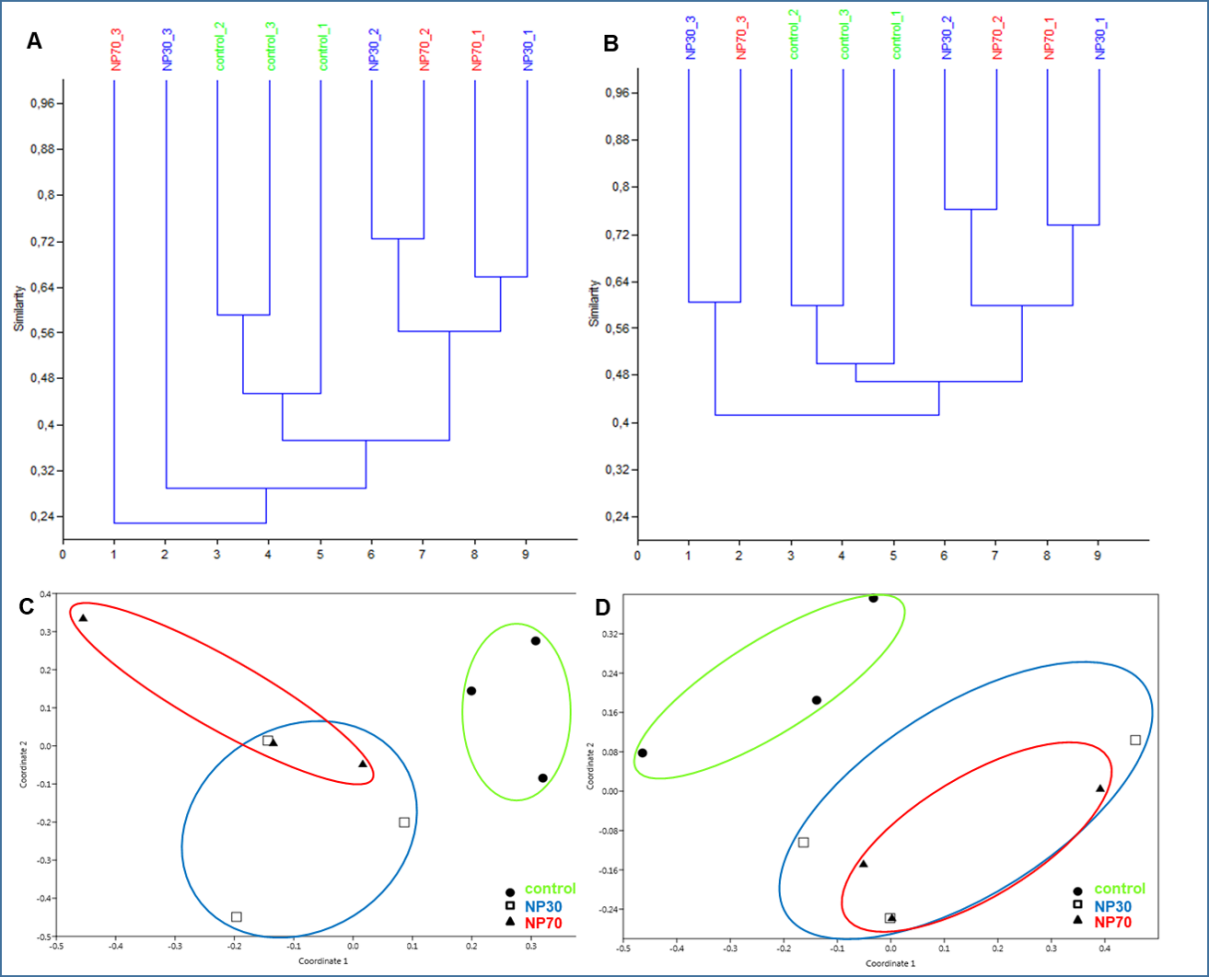
Cluster analysis. Cluster analysis based on the Bray Curtis index for *Msp*I (A) and *BstU*I (B) and the corresponding MDS plot for *Msp*I (C) and *BstU*I (D).

It is evident from cluster analysis and the MDS plot that the control biofilms formed a distinct group among each other, with similarities from 46 to 64% for *Msp*I and 50 to 60% for *BstU*I, and were clearly separated from NP30- and NP70-treated biofilms (Fig 3). NP30- and NP70-treated biofilms of replicate 1 and 2 showed high similarities among each other, of up to 72% (*Msp*I) and 77% (*BstU*I) (Fig 3A and 3B). Based on similar results obtained for *Msp*I and *BstU*I, the reproducibility of the data can be assumed.

### 3.3 Phylogenetic bacterial community composition based on clone libraries

**Fig 4:**
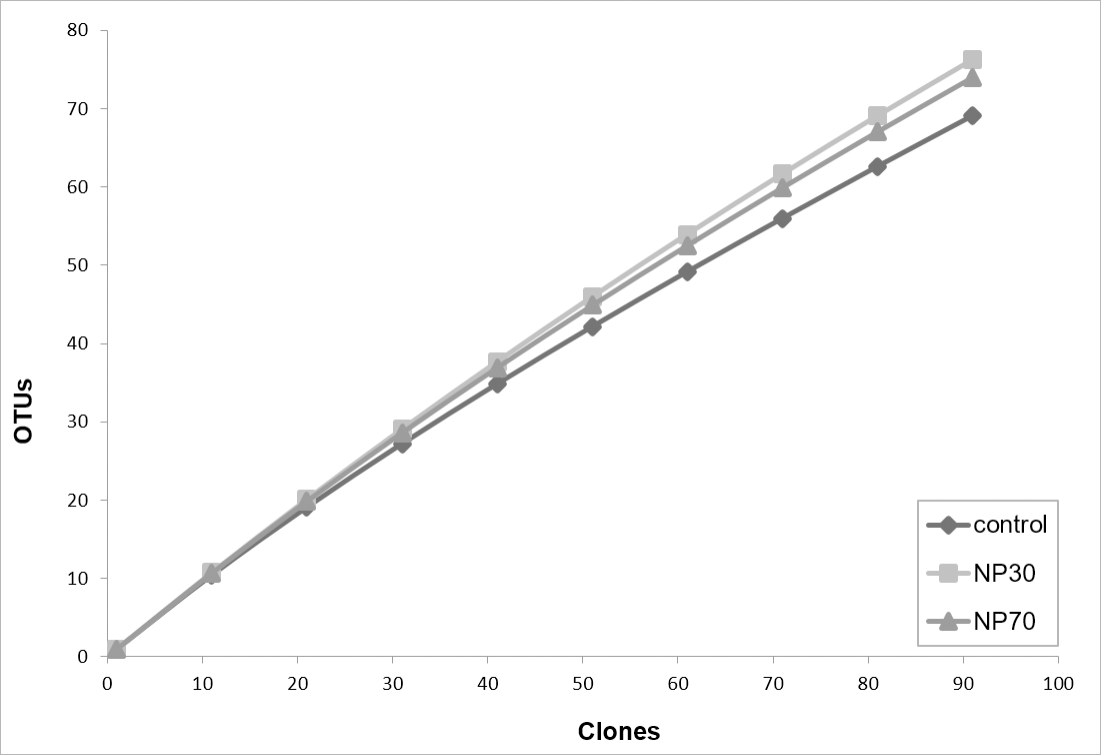
Diversity richness of biofilms. Diversity richness of biofilms simulated by rarefaction calculation based on the ARDRA patterns.

Analysis of the ARDRA patterns showed a high degree of phylogenetic heterogeneity for all biofilms because biofilms from all treatments (control, NP30, NP70) shared only 8 OTUs from a total of 226 OTUs among each other, whereas NP30- and NP70-treated biofilms had 17 OTUs in common. The biofilms included 71, 79, and 76 OTUs for the control, NP30-, and NP70-treated biofilms, respectively, which in accordance with the t-RFLP data gives an indication of a slightly greater diversity richness of the bacterial community in NP30- and NP70-treated biofilms. The diversity richness, calculated by rarefaction analysis (Fig 4) and further assessed by the Shannon, Alpha and Simpson diversity indices (S2 Table), indicated a relatively high degree of diversity, as already suggested by analysis of the t-RFLP profiles.

**Fig 5:**
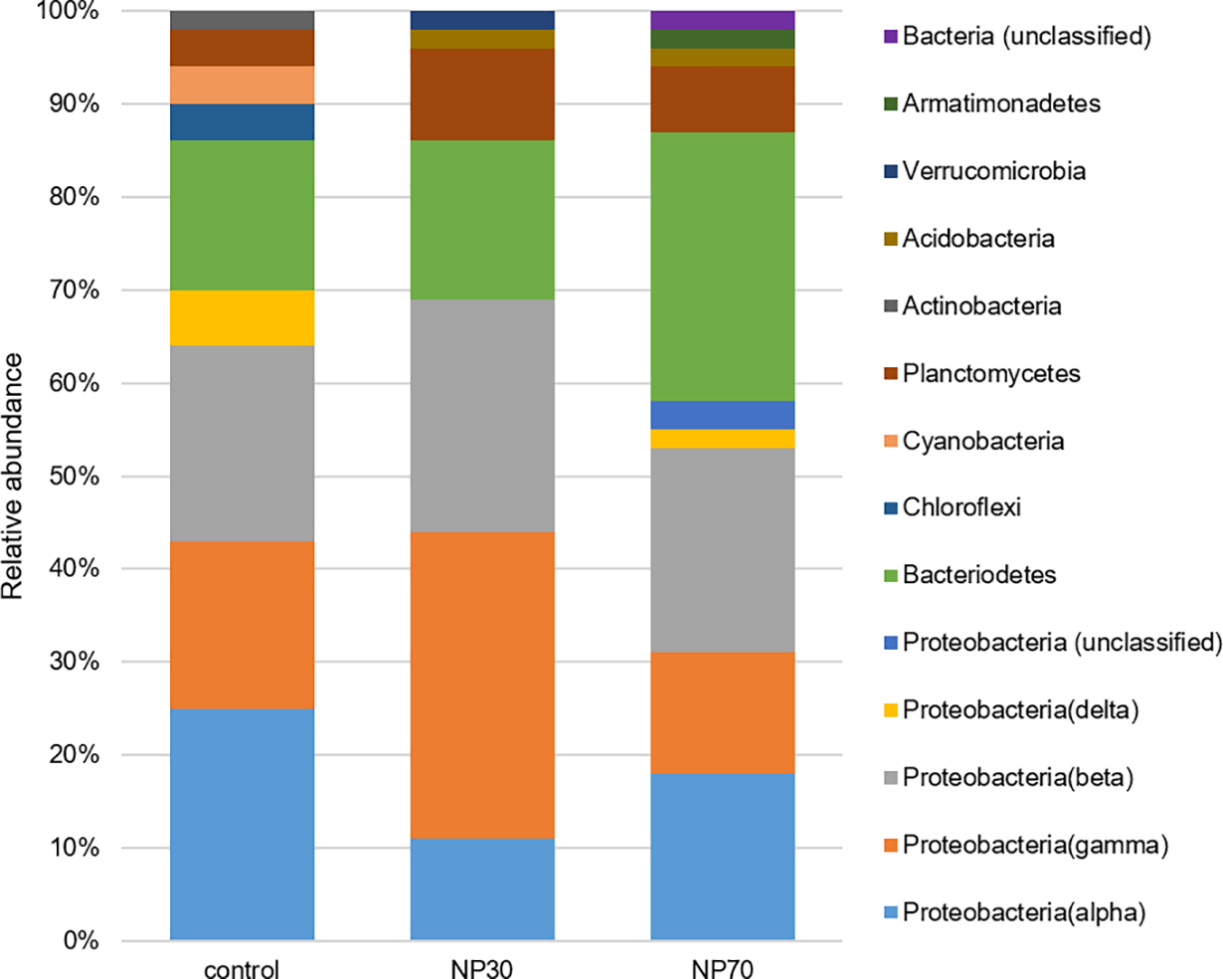
Distribution of OTUs. Distribution of OTUs at the phyla level for each treatment (control, NP30, NP70).

NP30- and NP70-treated biofilms differed from control biofilms by composition of phyla (Fig 5). NP30- and NP70-treated biofilms share 6 phyla among each other, whereas the control and treated biofilms have 5 phyla in common (Fig 5). All biofilm samples were predominantly composed of members of Proteobacteria (58–70%) and Bacteroidetes (16–29%). Actinobacteria, Chloroflexi, and Cyanobacteria were shown in lower abundances, ranging from 2–4% in the control biofilms only; members of Acidobacteria (2%) were unique to NP30- and NP70-treated biofilms. Apart from the other biofilm communities, NP70-treated biofilms hosted Armatimonadetes and NP30-treated biofilms hosted Verrucomicrobia, with 2% abundance, respectively.

**Fig 6:**
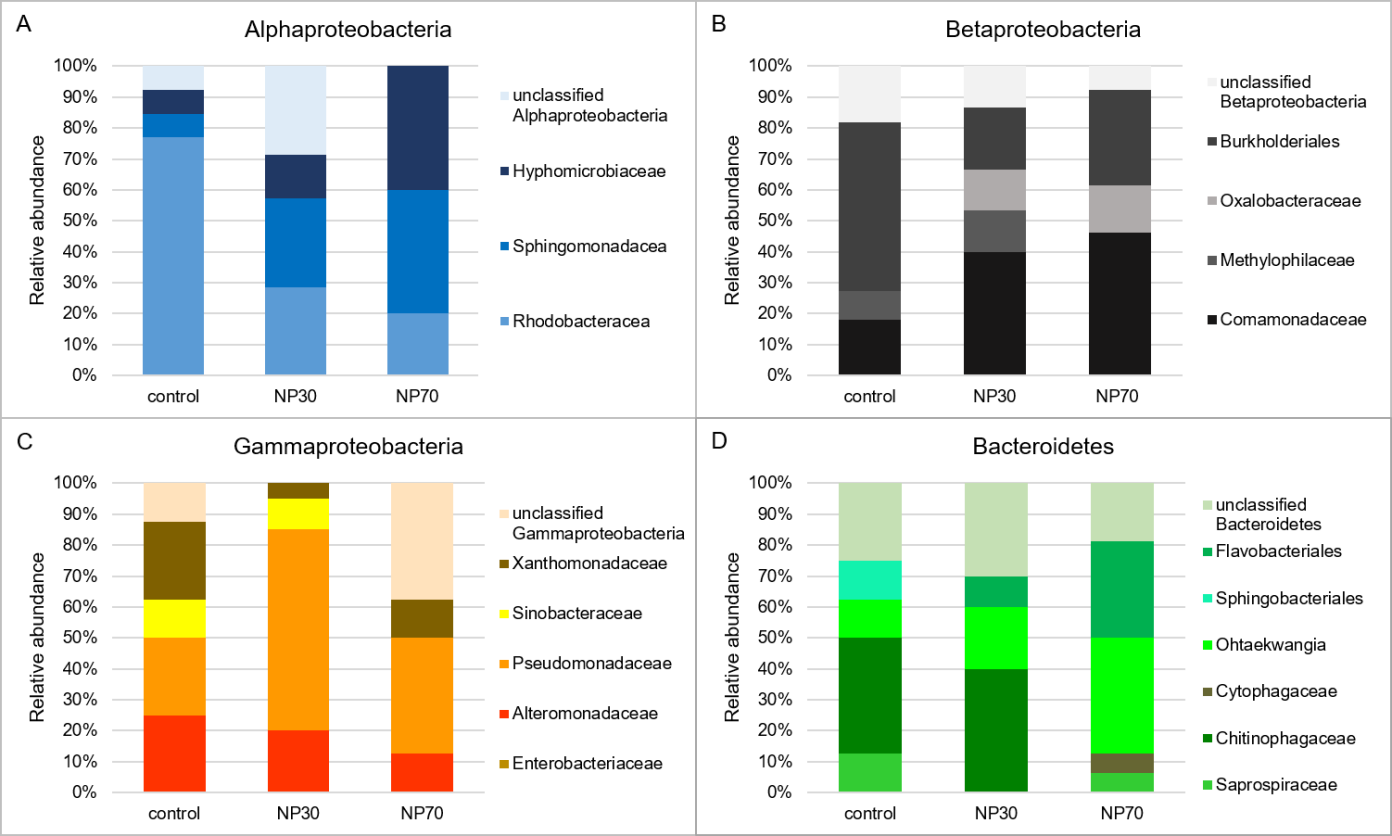
Relative abndance of OTUs. Relative abundance of OTUs for Alphaproteobacteria (A), Betaproteobacteria (B), Gammaproteobacteria (C), and Bacteriodetes (D) at the levels of order and family for each treatment (control, NP30, NP70).

Considerable differences in the community composition occurred between control biofilms and biofilms exposed to NP30 and NP70 at the lower phylogenetic levels and were most pronounced and abundant within the classes of Alphaproteobacteria, Betaproteobacteria, Gammaproteobacteria, and Bacteroidetes (Fig 6). The untreated biofilm community was dominated by Alphaproteobacteria, with 25% abundance. Exposure to NP30 or NP70 led to a remarkable reduction in the alphaproteobacterial abundance, between 11% and 18% (Fig 5). Rhodobacteraceae, with 77% abundance, were characteristic of Alphaproteobacteria in control biofilms, whereas Sphingomonadaceae and Hyphomicrobiaceae formed a major portion of NP30 and NP70 treated biofilms (Fig 6A).

While the community composition of all of the observed biofilms remained most stable within Betaproteobacteria at the class level (Fig 5), subdividing into lower taxonomic levels, such as the order and family, revealed that the family of Comamonadaceae was a major component for both treated biofilms, with 40% and 46% abundance for NP30 and NP70, respectively (Fig 6B). Within Gammaproteobacteria, biofilms exposed to NP30 and NP70 exhibited a high proportion of Pseudomonadaceae, which was most pronounced in NP30 treated biofilms, with 65% abundance (Fig 6C). Within Bacteroidetes, the highest heterogeneity in community composition developed due to NP70 treatment, as characterized by an abundance of 31% Flavobacteriales and the unique occurrence of 6% Cytophagaceae (Fig 6D). Phylogenetic analysis showed that the bacterial communities were strikingly distinct at lower phylogenetic levels, such as the order and family levels. Hence, data obtained from sequence analysis of cloned 16S rRNA genes support the assumption that exposure of biofilms to AgNPs forced the formation of bacterial community compositions distinct from control biofilms.

## 4 Discussion

Antibacterial activities of NPs depend on their properties, on the speciation of Ag as well as on the type of bacteria investigated [39]. AgNPs undergo complex and highly dynamic physical, chemical and biological transformations in aquatic environments, which alter their interactions with biota and further influence their intrinsic toxicity [1].

For instance, dissolution of AgNPs is influenced by the presence of dissolved oxygen [40–41], pH value and NOM [40]. We have recently shown that release of Ag^+^ ions by oxidative dissolution from citrate-stabilized AgNPs in the same size ranges was low or negligible in SAM-5S (pH 7.8; 5.06 mmol l^-1^) and R2A medium (pH 7.2; 8.75 mmol l^-1^) what are in the range of pH and ionic strength of water from the river Rhine (pH 7.4–8.3; 5–10 mmol l^-1^) (S1 Table) [22, 42]. Furthermore, reducing sugars within the extracellular polymeric substances (EPS) [43], Ag^+^ chelating compounds, either dissolved in the aqueous phase or as functional groups of EPS [44], and the coating of AgNPs with sodium citrate antagonize the dissolution of the Ag^+^ ions from AgNPs [45, 22]. Consequently, a rather negligible dissolution of AgNPs within the mesocosms is anticipated. Nevertheless, AgNPs i) may serve as a continual source of Ag^+^ ions particulary over long periods [46] and ii) produce reactive oxygen species in the oxidation process, which also have been reported to cause negative effects on microorganisms [47].

Aggregation of nanoparticles is induced predominantly by the presence and concentration of Ca^2+^ and Mg^2+^ ions which are common and essential components of freshwater environments [1, 42]. The results of our earlier work showed that the citrate-stabilized AgNPs are rapidly aggregated in water from river Rhine due to the presence of Ca^2+^ ions [7]. The aggregation of citrate-stabilized AgNPs in water from river Rhine was observed for a broad range of nanoparticle concentrations (ranging from 10 μg l^-1^ to 10 mg l^-1^) using two different techniques: dynamic light scattering (DLS) and hydrodynamic chromatography (HDC) coupled with the inductively coupled plasma mass spectrometer (ICP-MS) [7]. In addition to the size the HDC-ICP-MS method allows the determination of chemical composition of nanoparticle aggregates. Aggregation masks the differences between primary particle sizes and therefore, the differences in biological impact is less expected.

It is still under discussion whether the toxicity of AgNPs is attributed to additional “particle-specific” effects besides the antibacterial properties of released Ag^+^ ions [11]. The silver speciation rather than its total concentration influences bioavailability and is consistently a key parameter for understanding (eco)-toxicity [10]. Consequently, in order to meet the studies’ objectives i.e. to investigate the impact of AgNPs on the structure and bacterial community composition of freshwater biofilms it was not within the scope to measure the total Ag content within the biofilm material. Nevertheless, it has recently been shown that citrate-stabilized AgNPs in comparable size ranges undergo rapid aggregation in the Rhine river matrix reaching the aggregate size of 4 μm after 24 h exposure time [7] as well as accumulate within bacterial biofilms [22]. In this context, we assume that aggregation and subsequent sedimentation must have led to the accumulation of the AgNPs within the observed biofilms, which is in line with other studies, in which sedimentation of AgNPs on marine biofilms [14], estuarine assemblages [15], and periphyton [8] were demonstrated by measuring the total Ag content in the samples.

Analysis of clone libraries and t-RFLP profiling indicated a high degree of bacterial diversity in biofilms, which correlates with the level of diversity known from various natural environments. However, the rarefaction calculation also revealed that a higher number of cloned 16S rRNA genes should have been consulted to reach saturation in the rarefaction curve shape. Hence, to capture a higher number of OTUs, further work should combine 16S rRNA gene sequencing with a high-throughput method, such as 16S rRNA pyrosequencing, even though Sanger sequencing has more discriminatory power than pyrosequencing [48].

In our study, neither the diversity of the bacterial community nor the biomass parameters were impacted negatively, but the bacterial community composition was clearly affected and changed due to AgNP exposure. These findings are in good accordance to the unchanged bacterial diversity of marine biofilms after AgNP treatment [15]. However, confrontation of marine biofilms with AgNPs led to shifts in bacterial community composition [14], which is analogous to our results showing an altered composition of the bacterial biofilm community mediated by AgNP treatment. Change of the bacterial community composition and simultaneously unchanged diversity were also reported for the environmentally relevant heavy metals copper [49] and nickel [50]. Altogether, the unaffected architectural and biomass parameters of the biofilms in our study indicate a stable overall structural composition as well as continued biomass growth of the biofilms in the presence of AgNPs. Maintenance of structural properties even under an altered community composition indicates a particular resilient behavior of biofilms. A specific response to environmental changes by species contributing to the same ecosystem function [51] is a characteristic trait of that resilience behavior. The results of the mesocosm study presented here indicate that AgNP-sensitive bacteria seem to be replaced by more AgNP-tolerant species. Though this altered community composition ensured the maintenance of the same ecosystem function pertaining to the structural properties of the biofilm, other functions, such as bioremediation, might be potentially affected, as discussed in the following.

In our study, the two applied size fractions of AgNPs exhibit similar alterations in biofilm community composition independent of their sizes. This can be explained by aggregation of the AgNPs in water from river Rhine due to the presence of Ca^2+^ ions. Aggregation masks the differences between primary particle sizes of the two applied size fractions of AgNPs as discussed above. As shown by t-RFLP profiling and 16S rRNA gene library analysis, various bacterial responses were produced at the biofilm community level due to AgNP treatment. The shift in community composition was characterized by a numerical loss of bacterial taxa and simultaneous emergence of other bacterial entities.

Bacterial communities play a key role in the provision of enzymes and catalysis of chemical reactions. In this context, a probable loss of certain bacterial taxa as demonstrated in our study might trigger a potential impairment in the ecosystem function of biofilms such as NOM degradation and nutrient cycling.

The absence of Actinobacteria, Chloroflexi, and Cyanobacteria in both treated biofilms define certain species of this phyla as most susceptible to AgNPs because these phyla were unique for the untreated control biofilm. Actinobacteria are known for their variable metabolic properties, such as the production of secondary metabolites [52], which may serve as potent antibiotics [53]. Their absence in AgNP-containing freshwater biofilms might lead to a shrinking of antibiotic resources with a potential loss of defense strategies and adverse consequences for environmental health. Furthermore, native biofilms were dominated by Alphaproteobacteria, which is commonly a numerically dominant class of lotic biofilms [54]. Because members of Alphaproteobacteria, which are abundant in freshwater ecosystems, are capable of degrading complex organic compounds [55], the functional properties of biodegradation with respect to nutrient loads as well as transformation and/or degradation of pollutants might be impoverished in biofilms exposed to AgNPs. Furthermore, the ability of members of Alphaproteobacteria to be resistant to grazing [54] might contribute to the preservation of the overall biofilm architecture. Because the biofilm architecture is relevant for the biostabilization of cohesive sediments in aquatic habitats [56], this important ecosystem service provided by biofilms might be affected in AgNP-treated biofilms, which are characterized by a lower abundance of Alphaproteobacteria.

Bacterial biofilm communities in treated biofilms showed a higher degree of phylogenetic diversity compared to untreated biofilms, as demonstrated by the emergence of additional bacterial taxa. The increase of Acidobacteria abundance, shown for NP30- and NP70- treated biofilms, accompanied by adverse effects on the abundance of Chloroflexi in AgNP-treated biofilms, has also been reported for bacterial communities developed under AgNP exposition in activated sludge [17]. Acidobacteria might exhibit silver resistance mechanisms, such as drug reporters [57], and silver-sensitive Chloroflexi might be associated with a lack of lipid outer membrane and specialized secretion systems (e.g., type I, II and III secretion systems) [17]. Furthermore, the community is distinctly shaped to taxa that have been reported in heavy metal-polluted environments, such as Sphingomonadales and Comamonadaceae [58–63]. Comamonadaceae, which are predominant inhabitants of treated biofilms, are often associated with heavy metal-polluted environments [58–61]. The genus *Curvibacter*, observed in NP30-treated biofilms, also appeared under exposure to Cr(III) and Pb(II) [58]. Sphingomonadales, which formed a major part of Alphaproteobacteria in NP70-treated biofilms, have been reported in a nickel-polluted river [62] and a Cu(II)-removing biofilm [63]. Of particular note is that Gammaproteobacterial OTUs in NP30-treated biofilms are mainly related to the species *Pseudomonas putida*, an ubiquitous saprophytic bacterium with heavy metal tolerance mechanisms that thrives in environments with metal(oid) contamination [64–65]. In sum, our findings suggest that adverse conditions induced by heavy metals and AgNPs might be alike and that analogous selective pressure prevails.

The bacterial biofilm composition is a multispecies community, which might include the entire range from susceptibility towards silver to tolerance against silver. As discussed above, the absence of Chloroflexi might them define as silver-sensitive. Contrary, Acidobacteria and Comamonadaceae, as members of treated biofilms, the genus *Curvibacter* and the species *Pseudomonas putida*, observed in NP30-treated biofilms, and Sphingomonadales in the NP70-treated biofilms seem to be silver-tolerant. Thus, tolerance mechanisms against silver stress can be assumed. Furthermore, the maintenance of the structural properties of the treated biofilms even under this altered community composition indicates a particular resilient behavior. These findings form an integral part of the biofilm’s response to the treatment with AgNPs including silver-sensitive and silver-tolerant species towards a rather low concentration of 600 μg l^-1^. It is therefore reasonable to assume that a unique lethal concentration of AgNPs can be assigned to each taxa and even each species. Therefore, this specialized biofilm community might be accompanied by a loss of metabolic and ecophysiological capabilities, putatively impairing ecosystem functions, even in a partly sublethal concentration of 600 μg l^-1^. A concentration of 600 μg l^-1^ AgNPs would mimic a worst case scenario, such as the resuspension of sediments by flooding events or production plant outfalls and would be still at the higher end of the concentration range for AgNPs estimated for natural environments [3]. Because of biofilm-mediated ecological services, the replacement of AgNP-sensitive species by more AgNP-tolerant species raises environmental concerns regarding the release of AgNPs into freshwater systems.

## 5 Conclusions

Similarity between the treated biofilms and their replicates as well as relative homogeneity and similarity between the replicates of control biofilms suggest that the design of the mesocosm experiment was appropriate to mirror a freshwater biofilm ecosystem close to natural conditions. Furthermore, we proofed a bacterial community composition which reflects the bacterial community composition commonly found in stream biofilm communities [54]. Because we applied several approaches to analyze biofilms as a whole in response to AgNP treatment, we found that unchanged biomass parameters were accompanied by a change in bacterial diversity, which was not diminished but rather increased by AgNPs. Consequently, insights into the resilience of the biofilms in response to a pollutant stress were obtained. Although the structural properties of the biofilms were maintained, a considerable displacement of bacterial taxa within the bacterial community occurred after exposure to a single pulse addition of AgNPs, as is believed to occur within flood events. These phylogenetic changes provided insights into biogeochemical impacts, which may lead to an impairment of ecosystem functions pertaining to biodegradation with respect to nutrient loads, transformation and/or degradation of pollutants, and biostabilization. We demonstrated that both sizes of AgNPs affected the bacterial community composition in a similar way, which is most likely attributed to the masking of differences between primary particle size due to rapid aggregation of AgNPs in river water observed in our earlier study [7].

The putatively AgNP-sensitive bacterial taxa Actinobacteria, Chloroflexi, and Cyanobacteria were displaced by the taxa Acidobacteria, Sphingomonadales, and Comamonadaceae. Comparable shifts within the community composition have been reported in the past for heavy metal-treated bacterial communities, which indicates analogous mechanisms of toxicity. These phylogenetic changes were induced by a rather low concentration of 600 μg l^-1^ AgNPs, which might threaten freshwater biofilms in the event of production plant outfalls. In sum, this shift in the community composition of bacterial freshwater biofilms may lead to an impairment of ecosystem functions pertaining to biodegradation with respect to nutrient loads, transformation and/or degradation of pollutants, and biostabilization.

## Supporting information

S1 Table Physico-chemical water parameters of mesocosm and aquaria. (DOCX)

S2 Table Estimation of diversity richness as assessed with Shannon, Alpha and Simpson diversity indices. (DOCX)
