# Supplementary material for: Effects of low dose silver nanoparticle treatment on the structure and community composition of bacterial freshwater biofilms
Source: PLoS One. 2018 Jun 14;13(6):e0199132. doi: 10.1371/journal.pone.0199132 (PMC6002094; doi:10.1371/journal.pone.0199132)
Supplement: S1 Table — (DOCX) [file pone.0199132.s001.docx]

**S1 Table**.

| **experiment** | **day** | **pH** | **conductivity**  **(µS cm^-1^)** | **O_2_**  **(%)** | **O_2_**  **(mg l^-1^)** | **flow velocity**  **(m s^-1^)** | **°C** | **NO_3_^-^**  **(mg l^-1^)** | **NO_2_^-^**  **(mg l^-1^)** | **NH_4_^+^**  **(mg l^-1^)** | **PO_4_^3-^**  **(mg l^-1^)** |
| --- | --- | --- | --- | --- | --- | --- | --- | --- | --- | --- | --- |
| ***1^st^ replicate*** |  |  |  |  |  |  |  |  |  |  |  |
| mesocosm | 0/14 | 8.2 | 714 | 101 | 9.9 | 0.04 – 0.2 | 16 | < 0.5 | < 3 | < 4 | < 0.05 |
| control | 0 | 8.2 | 718 | 100 | 9.1 | 0.04 – 0.3 | 20 | < 0.5 | < 3 | < 4 | < 0.05 |
| NP30 | 0 | 8.2 | 715 | 100 | 9.1 | 0.04 – 0.3 | 20 | < 0.5 | < 3 | < 4 | < 0.05 |
| NP70 | 0 | 8.2 | 713 | 100 | 9.1 | 0.04 – 0.3 | 20 | < 0.5 | < 3 | < 4 | < 0.05 |
| mesocosm | 7 | 7.6 | 712 | n.d.^a^ | n.d.^a^ | 0.04 – 0.2 | 15 | < 0.5 | < 3 | < 4 | < 0.05 |
| control | 7 | 7.7 | 746 | n.d.^a^ | n.d.^a^ | 0.04 – 0.3 | 20 | < 0.5 | < 3 | < 4 | < 0.05 |
| NP30 | 7 | 8.3 | 730 | n.d.^a^ | n.d.^a^ | 0.04 – 0.3 | 20 | < 0.5 | < 3 | < 4 | < 0.05 |
| NP70 | 7 | 8.3 | 743 | n.d.^a^ | n.d.^a^ | 0.04 – 0.3 | 20 | < 0.5 | < 3 | < 4 | < 0.05 |
| mesocosm | 14 | 8.2 | 775 | 101 | 10.1 | 0.04 – 0.2 | 15 | < 0.5 | < 3 | < 4 | < 0.05 |
| control | 14 | 8.3 | 848 | 104 | 9.3 | 0.04 – 0.3 | 19 | < 0.5 | < 3 | < 4 | < 0.05 |
| NP30 | 14 | 8.3 | 819 | 101 | 9.2 | 0.04 – 0.3 | 19 | < 0.5 | < 3 | < 4 | < 0.05 |
| NP70 | 14 | 8.3 | 841 | 101 | 9.2 | 0.04 – 0.3 | 19 | < 0.5 | < 3 | < 4 | < 0.05 |
| ***2^nd^ replicate*** |  |  |  |  |  |  |  |  |  |  |  |
| mesocosm | 0 | 8.2 | 712 | 100 | 10 | 0.04 – 0.2 | 16 | < 0.5 | < 3 | < 4 | < 0.05 |
| control | 0 | 8.3 | 713 | 100 | 9.9 | 0.04 – 0.3 | 16 | < 0.5 | < 3 | < 4 | < 0.05 |
| NP30 | 0 | 7.8 | 713 | 100 | 9.9 | 0.04 – 0.3 | 16 | < 0.5 | < 3 | < 4 | < 0.05 |
| NP70 | 0 | 7.9 | 712 | 101 | 9.9 | 0.04 – 0.3 | 16 | < 0.5 | < 3 | < 4 | < 0.05 |
| mesocosm | 14 | 8.1 | 668 | n.d.^a^ | n.d.^a^ | 0.04 – 0.2 | 15 | < 0.5 | < 3 | < 4 | < 0.05 |
| control | 14 | 8.1 | 672 | n.d.^a^ | n.d.^a^ | 0.04 – 0.3 | 17 | < 0.5 | < 3 | < 4 | < 0.05 |
| NP30 | 14 | 8.1 | 675 | n.d.^a^ | n.d.^a^ | 0.04 – 0.3 | 17 | < 0.5 | < 3 | < 4 | < 0.05 |
| NP70 | 14 | 8.1 | 673 | n.d.^a^ | n.d.^a^ | 0.04 – 0.3 | 17 | < 0.5 | < 3 | < 4 | < 0.05 |
| ***3^rd^ replicate*** |  |  |  |  |  |  |  |  |  |  |  |
| mesocosm | - | 8.3 | 676 | n.d.^a^ | n.d.^a^ | 0.04 – 0.2 | 15 | < 0.5 | < 3 | < 4 | < 0.05 |
| control | 0 | 8.2 | 684 | n.d.^a^ | n.d.^a^ | 0.04 – 0.3 | 20 | < 0.5 | < 3 | < 4 | < 0.05 |
| NP30 | 0 | 8.2 | 688 | n.d.^a^ | n.d.^a^ | 0.04 – 0.3 | 20 | < 0.5 | < 3 | < 4 | < 0.05 |
| NP70 | 0 | 8.2 | 687 | n.d.^a^ | n.d.^a^ | 0.04 – 0.3 | 20 | < 0.5 | < 3 | < 4 | < 0.05 |
| mesocosm | - | 7.8 | 720 | n.d.^a^ | n.d.^a^ | 0.04 – 0.2 | 17 | < 0.5 | < 3 | < 4 | < 0.05 |
| control | 14 | 7.4 | 706 | n.d.^a^ | n.d.^a^ | 0.04 – 0.3 | 20 | < 0.5 | < 3 | < 4 | < 0.05 |
| NP30 | 14 | 7.6 | 665 | n.d.^a^ | n.d.^a^ | 0.04 – 0.3 | 20 | < 0.5 | < 3 | < 4 | < 0.05 |
| NP70 | 14 | 7.6 | 671 | n.d.^a^ | n.d.^a^ | 0.04 – 0.3 | 20 | < 0.5 | < 3 | < 4 | < 0.05 |

^a^ n.d. not determined
