# Supplementary material for: Effects of low dose silver nanoparticle treatment on the structure and community composition of bacterial freshwater biofilms
Source: PLoS One. 2018 Jun 14;13(6):e0199132. doi: 10.1371/journal.pone.0199132 (PMC6002094; doi:10.1371/journal.pone.0199132)
Supplement: S2 Table — (DOCX) [file pone.0199132.s002.docx]

**S2 Table**

|  | **control** | **NP30** | **NP70** |
| --- | --- | --- | --- |
| **Shannon H’ log base 10** | 1.78 | 1.87 | 1.84 |
| **Shannon Hmax log base 10** | 1.85 | 1.90 | 1.88 |
| **Shannon J’** | 0.96 | 0.98 | 0.98 |
| **Alpha** | 132.12 | 220.48 | 184.83 |
| **Simpson diversity (1/D)** | 93 | 223.25 | 174.84 |
